# Microencapsulation of Bioactive Ingredients for Their Delivery into Fermented Milk Products: A Review

**DOI:** 10.3390/molecules26154601

**Published:** 2021-07-29

**Authors:** Ruta Gruskiene, Alma Bockuviene, Jolanta Sereikaite

**Affiliations:** 1 Department of Chemistry and Bioengineering, Vilnius Gediminas Technical University, 10223 Vilnius, Lithuania; ruta.gruskiene@vgtu.lt; 2 Department of Polymer Chemistry, Institute of Chemistry, Vilnius University, 01513 Vilnius, Lithuania; burvytealma@gmail.com

**Keywords:** encapsulation, encapsulant material, yogurt, cheese, kefir, carotenoids, phenolics, probiotics, omega-3, micronutrients

## Abstract

The popularity and consumption of fermented milk products are growing. On the other hand, consumers are interested in health-promoting and functional foods. Fermented milk products are an excellent matrix for the incorporation of bioactive ingredients, making them functional foods. To overcome the instability or low solubility of many bioactive ingredients under various environmental conditions, the encapsulation approach was developed. This review analyzes the fortification of three fermented milk products, i.e., yogurt, cheese, and kefir with bioactive ingredients. The encapsulation methods and techniques alongside the encapsulant materials for carotenoids, phenolic compounds, omega-3, probiotics, and other micronutrients are discussed. The effect of encapsulation on the properties of bioactive ingredients themselves and on textural and sensory properties of fermented milk products is also presented.

## 1. Introduction

Nowadays, there is a growing consumer interest in products that promote health and well-being. The beneficial effect of fermented milk products has been well-known since ancient times. Their health benefits are attributed to the action and metabolites of lactic acid bacteria (LAB) as well as biologically active components of milk. In addition to LAB, the diverse set of microorganisms, including yeast, molds, and bacteria is used for the production of fermented milk products [1]. They can be divided into two large groups, i.e., fermented milks and cheeses. There is a great variety of fermented milks depending on a specific culture used for the fermentation, the type of raw material (cow, sheep, goat, mare, camel, or buffalo milk), and the geographic location [2]. Yogurt obtained by symbiotic cultures of *Streptococcus thermophilus* and *Lactobacillus delbrueckii* subsp. *bulgaricus* is the most popular fermented milk product all over the world. Yogurt based on *Streptococcus thermophilus* and all species of *Lactobacillus*, acidophilus milk, kefir, kumys, and cultured buttermilk are also known and popular among consumers [2,3]. Cheeses exhibit a very high diversity of composition and structure. According to the FAO/WHO Codex Alimentarius Commission, they are classified depending on the water content in the fat-free matter, fat content in the dry matter, and ripening [1]. Fermented milk products modulate the intestinal microbiota and suppress the growth of pathogens due to the action of metabolites, such as lactic acid and bacteriocins accumulated during the fermentation process. There are data that the consumption of fermented milk products is associated with a lower risk of developing stroke, cardiovascular disease, and type 2 diabetes mellitus [3,4]. Recently, the possible anti-cancer effect of kefir, especially in the case of intestinal and colorectal cancer, has been announced [5].

**Figure 1:**
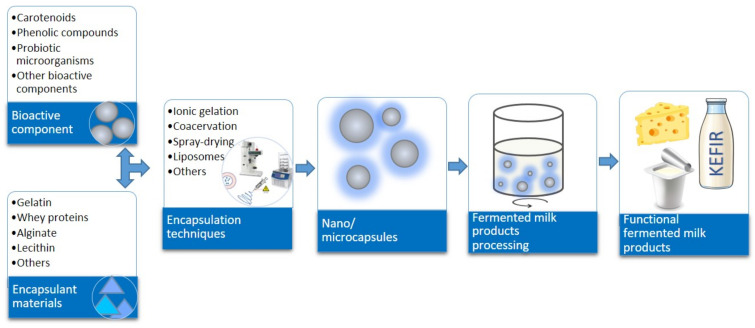
Schematic representation of the delivery of encapsulated bioactive ingredients into fermented milk products.

Despite their own health benefits, fermented milk products are an excellent matrix for the incorporation of bioactive ingredients, making them true functional foods [3]. To provide functional characteristics, bioactive compounds and probiotics are largely investigated. However, many of them are unstable under the changes of various environmental conditions such as temperature, pH, and light; exhibit a residual taste, limiting their application; or have low solubility [6]. To overcome these drawbacks, the strategy of nano/microencapsulation of bioactive ingredients was developed (Figure 1). Encapsulation is the method when an active agent is entrapped within another substance. The active agent can be also called the core or payload. The encapsulating substance can be termed the shell, wall, matrix, encapsulant, or carrier. The active compound can be found in the interior of the shell-like structure of the encapsulating material or can be interspersed within the matrix. The carrier materials act as a physicochemical barrier against environmental conditions and improve the physicochemical and biological characteristics of the encapsulated active agents. The dimensions of encapsulated product can be at the scale of nano- and micrometers [7,8,9]. Nanoscale technology has the advantages compared to microencapsulation. Nanocapsules have higher surface area. That may lead to the improvement in bioavailability and solubility of bioactive ingredients. Moreover, lower scale capsules are more preferable in terms of sensory properties of food products. As an example, nanoemulsions having the size of droplets less than 100 nm are optically transparent. That is very important for the application in the beverage industry [10,11].

The choice of encapsulation techniques and delivery system depends not only on the physical and chemical properties of the bioactive compounds but also on the end product characteristics. Encapsulated and delivered bioactive compounds have to not alter undesirably the appearance, texture, or mouthfeel of the end product. Delivery systems of bioactive compounds have to be easily scaled up, economic, and prepared from ingredients that are acceptable in foods [12,13].

Herein, we review the nano/micro encapsulation methods and materials used for the delivery of bioactive ingredients into the most popular fermented milk products, i.e., yogurt, kefir, and cheese. The effect of encapsulated ingredients on the health-promoting and sensory properties of products is discussed as well.

## 2. Yogurt Fortification with Nano/Microencapsulated Bioactive Ingredients

### 2.1. Carotenoids and Carotenoids Containing Components

Carotenoids are a group of isoprenoid compounds biosynthesized by all photosynthetic organisms and many bacteria and fungi. They are natural pigments and have an intense red/orange/yellow color. Most animals (including humans) are unable to synthesize carotenoids in their bodies. Humans obtain carotenoids from the diet mainly from fruits and vegetable-derived foods. The consumption of carotenoids is associated with health benefits, i.e., the lower risk of cancer, cardiovascular diseases, age-related macular degeneration and type 2 diabetes, and the enhanced immune system function. Moreover, some of them have provitamin A activity. Therefore, the normal daily dietary intake of carotenoids is essential having in mind their role in retaining human body function [14,15,16]. In their structure, carotenoids have a polyene system containing conjugated double bonds and can act as single oxygen quenchers and free radical scavengers. However, due to the double bonds in their structure, carotenoids are susceptible to heat, oxygen, and temperature, and easily undergo the isomerization and degradation [17].

The fortification of foods with carotenoids, including yogurt, can ensure their daily intake. Table 1 summarizes recently published findings concerning carotenoids delivery systems into yogurt [18,19,20,21,22,23,24,25,26,27,28,29,30]. As seen, pure carotenoids or the extracts from fruits and vegetables are used for encapsulation. Palm oil rich in carotenoids can also be encapsulated and entrapped into yogurt. Shrimp waste can serve as a source of astaxanthin for the application in the dairy industry. In crustaceans, carotenoids occur due to their absorption from the diet, and astaxanthin is the principal one [31]. As seen from Table 1, various encapsulation systems are used, i.e., emulsion-based, including solid lipid nanoparticles, biopolymer-based, including molecular inclusion complexes, surfactant-based (liposomes), and freeze- and spray-drying ones [19,20,21,22,23,24,25,26,27,28,29,30]. In addition, a modern electro-spinning technique was applied for the tomato peel extract encapsulation to use the prepared nanofibers in yogurt [18]. The encapsulation of pure carotenoid or their extracts ensures more stable yogurt coloration due to the higher carotenoid retention compared to the free form [20,21,22,23,28]. The addition of encapsulated carotenoids increases the functionality of yogurt, including the increase of their antioxidant activity [18]. However, there are only a few works, in which the release and bioaccessibility of encapsulated carotenoids added into yogurt are analyzed [25,29,30]. In general, the sensory acceptability of yogurt fortified with encapsulated carotenoids is similar or even higher compared to yogurt fortified with the free form of carotenoids or without fortification [18,19,23,24,26,28]. However, the consumers perceived the differences in the taste and mouthfeel of yogurt supplemented with astaxanthin-loaded alginate-chitosan beads although the appearance, color, and aroma of the yogurt were not changed [27].

### 2.2. Phenolic Compounds (Phenolics) Containing Components

Phenolics are a second large group of compounds delivered into yogurt in the encapsulated form. Phenolics are diverse in their structure and are classified into the following main classes, i.e., flavonoids, phenolic acids, coumarins, lignans, tannins, and stilbenes. They are secondary metabolites of plants, and usually, their extracts serve as a dietary source of phenolics. In recent years, they have received the attention due to the positive effects on human health. Phenolics have antioxidant, antimicrobial, and anti-inflammatory activities and show anticarcinogenic effects [31,32,33]. However, their instability in food, the bitter taste, and low bioavailability often limit their application.

The nano/microencapsulation approach facilitates the delivery of phenolics into various food matrices. For the encapsulation, mainly the extracts from various plants and fruits are used (Table 2). Surfactant-based encapsulation systems such as liposomes coated with chitosan or spray-dried coated liposomes and spray-drying method are preferred for yogurt fortification [34,35,36,37,38]. In addition, the particles are prepared by the coacervation [39], ionic gelation [40], and ultrasonication methods [41]. For yogurt enrichment with resveratrol, niosomes were evaluated [42]. These vesicles are formed by the self-assembly of non-ionic surfactants. Cholesterol is often used for their preparation. It is important for niosomes properties such as membrane permeability, rigidity, and encapsulation efficiency [43]. Since cholesterol has potential adverse health effects and is not suitable for functional foods, for resveratrol delivery, it was substituted by lauryl alcohol [42]. The addition of encapsulated extracts had no influence on the texture properties and color of yogurt independently on the encapsulation system. The antioxidant activity of yogurt increased or retained longer compared to the nonencapsulated form of the extracts [34,35,36,44].

### 2.3. Other Bioactive Components

Some publications presented research on yogurt fortification with vitamin D_3,_ which is fat-soluble and susceptible to the degradation by low pH, UV, and oxidation [46,47,48]. Vitamin D_3_ was delivered in the form of spray-dried nanoliposomes [49], nanocapsules based on the lipid carrier precirol [50], oil-in-water emulsion stabilized by whey protein or by whey protein plus carboxymethylcellulose [51], or encapsulated in re-assembled casein micelles [50]. Encapsulated vitamin D_3_ showed the high stability against deterioration during the shelf life of yogurt. Moreover, the investigation of bioavailability demonstrated that the delivery of vitamin D_3_ in the encapsulated form is feasible [51,52]. The addition of vitamin D_3_ increases the high nutritional value of yogurt, is beneficial for human health, and helps to overcome vitamin deficiency in the diet.

Attempts were made to entrap other micronutrients into yogurt. First, yogurt is considered as a suitable vehicle for iron because the iron-fortified dairy products have high iron bioavailability [53]. The encapsulation of iron is aimed at the elimination of the effect on taste, appearance, product stability, and lipid oxidation [54,55]. Iron as a core material is used in the form of ferrous sulfate, ferric ammonium sulfate, ferrous bisglycinate, and ferrous lactate. Usually, iron is encapsulated in the combination with vitamin C, which facilitates nonheme iron absorption and participates in its metabolism [56]. The microcapsules were prepared using vegetable fats or polyglycerol monostearate as a coating material [53,57]. The effect on the sensory properties of microcapsules carrying different iron salts was different. Yogurt fortified with ferrous bisglycinate and ferrous lactate had the lower acceptability in terms of texture, flavor, and appearance, while yogurt fortified with encapsulated ferrous sulfate was not statistically different from a control yogurt [57]. In the presence of iron in polyglycerol monostearate-based microcapsules, the lipid oxidation process was significantly slower as compared to nonencapsulated iron [53]. Recently, for yogurt fortification, iron-entrapped niosomes formed by the self-assembly of non-ionic surfactants in aqueous media were developed by the ethanol injection method [58]. They were found to be suitable for yogurt functionalization since they had no effect on the textural and rheological properties of yogurt. Moreover, addition experiments demonstrated that whey proteins stabilize niosomes.

Recently, a few reports were published studying the encapsulation of plant essential oils and their delivery into yogurt [59,60,61]. Their addition is beneficial due to their antimicrobial and antioxidant activities [62]. The encapsulation solves the problem of their stability and increases the value of fermented milk products. Yogurt supplemented with the essential oil of basil, mint, fennel, or lavender, which were encapsulated in sodium alginate, had better characteristics in terms of antioxidant activity compared to the control sample. The best effect was achieved by adding the encapsulated basil essential oil. The decrease of antioxidant activity after 20 days of storage was not large [60]. Essential oils are a source of flavoring agents. For the preparation of flavored yogurt, the microcapsules prepared by coating *Mellissa* oil with sodium caseinate and whey protein isolate were added. The encapsulation prevented the spontaneous release of essential oil, and the supplementation increased the antioxidant activity of products. The addition of microcapsules at the concentration of 0.75–1.5% (*w*/*v*) did not change the sensory properties of yogurt [59]. Garlic essential oil is known for its antibacterial activity against both Gram-positive and Gram-negative bacteria. It is rich in organosulfur compounds responsible for the antimicrobial properties. Allicin, which is very sensitive to high temperature and high pH values, is the major component of the garlic essential oil. The encapsulation can increase its stability and solubility in water-based foods. Moreover, the encapsulation suppresses the unpleasant and annoying smell of garlic essential oil. For that purpose, nanophytosomes containing the oil were developed, characterized, and used in yogurt. Nanophytosomes were obtained using phosphatidylcholine and fabricated by the thin-layer hydration method and following homogenization or probe sonication, or the combination of homogenization and probe sonication. The nanophytosomes showed stable antioxidant activity during the storage time of 30 days. The addition of nanophytosomes had no effect on the texture and color of the prepared yogurt. The difference in aroma between the control sample and the sample supplemented with the nanophytosomes carrying garlic essential oil was low [61].

Yogurt is considered as a particularly suitable food matrix for fortification with long-chain omega-3 polyunsaturated fatty acids (PUFA) due to its popularity among consumers and storage conditions at lower temperatures favorable for fatty acids stability. PUFA has health benefits including the prevention of cardiovascular diseases and reduction of inflammation. Moreover, PUFA positively affects cognitive health and age-related decline in muscle mass. In the diet, eicosapentaenoic acid (EPA) and docosahexaenoic acid (DHA), which are found in fish oil, are the most important [63]. To elevate the daily intake of PUFA and mask the undesirable sensory properties of fish oil, various encapsulation forms were developed for the delivery into yogurt. Micro- and double emulsions [64,65], nanoliposomes [66], and particles obtained by the coacervation method using gelatin/acacia gum or by the thermal polymerization of whey proteins were prepared [67,68]. The encapsulation protects unsaturated fatty acids from deterioration. The authors emphasize the reduction in peroxide value and the higher content of EPA and DHA compared to yogurt containing free fish oil after three weeks of storage. Overall, the sensory characteristics of yogurt enriched in encapsulated fish oil are acceptable [64,66,67]. However, depending on the encapsulation method and the amount of fish oil, an unfavorable fishy taste might be detected [65]. Fish oil can be incorporated into the nanoemulsion together with γ-oryzanol, which is a mixture of ferulic acid esters of triterpene alcohols and sterols. γ-Oryzanol is one of the major bioactive compounds in rice bran and has antioxidant and anti-inflammatory activity [69]. In yogurt, γ-oryzanol protects fish oil against deterioration acting as an antioxidant [70]. In addition to fish oil, algal oil can also serve as a source of DHA and can be a good alternative for vegetarians and vegans. Lane et al. showed that the consumption of yogurt enriched in algal oil nanoemulsions increased the bioavailability of DHA compared to bulk oil [71]. Sweet almond oil and sesame oil are also rich in mono- and polyunsaturated fatty acids. Recently, the investigation of the properties of yogurt enriched in nanoemulsions of those oils has been published. Fortified yogurt exhibited reduced syneresis, lower pH values, and increased antioxidant activity, since those oils contain various phytochemicals and antioxidants. Sensory analysis showed that yogurt enriched in sesame oil nanoemulsions had the highest score [72]. Screening of scientific papers shows that coencapsulation of bioactive components is a quite a scarce phenomenon. Comunian et al. coencapsulated echium oil rich in polyunsaturated fatty acids, especially stearidonic acid and a mixture of phytosterols and sinapic acid known as an antioxidant. Microcapsules were obtained by the complex coacervation method using gelatin and arabic gum or cashew gum as wall materials. The encapsulation provided the stability of bioactive components and their release in gastric and intestinal fluids. The fortification of yogurt with microcapsules did not change its physicochemical, rheological, and sensorial properties [73].

In addition, to functionalize yogurt, encapsulation systems for purple rice bran oil rich in α-tocopherol, γ-tocotrienol, and γ-oryzanol were developed based on nanoemulsions [74], and for *Nigella sativa* oil containing thymoquinone [75]. The latter was emulsified and spray-dried using modified starch and maltodextrin as wall materials. The encapsulation of a palm tocotrienol rich fraction in the form of alginate–chitosan microcapsules increased the stability of tocotrienols, especially when the microcapsules were incorporated into yogurt [76]. The extract of mushroom *Agaricus bisporus*, which is largely used over the world, contains bioactive compounds such as ergosterol and mycosterol, exhibiting beneficial properties for human health. The extract was encapsulated by spray drying using maltodextrin crosslinked with citric acid as a carrier material and was found to be a promising bioactive ingredient for yogurt functionalization [77]. For the dairy products fortification with microalgae, *Spirulina*-loaded alginate microcapsules coated with whey protein concentrate were prepared. Microencapsulation improved the acceptance of fortified yogurt, making the color of the final product lighter [78]. 

### 2.4. Probiotic Microorganisms

*Streptococcus thermophilus* and *Lactobacillus delbrueckii* subsp. *bulgaricus* as starter cultures of yogurts are considered to have health benefits. However, they are not natural inhabitants of the intestine. To increase yogurt functionality, it can be supplemented with probiotic microorganisms. The genera of *Lactobacillus* and *Bifidobacterium* are the most studied microorganisms for functional food production. However, most of them show the low tolerance to acidic and aerated media. Moreover, the number of probiotic microorganisms decreases due to the action of gastric juice and bile salts [79]. To exhibit therapeutic benefits, products have to contain at least 10^6^ live microorganisms per g or mL of the product [80]. To increase the viability of probiotic microorganisms, the encapsulation approach has been considered as a way to solve this problem. The encapsulation of bifidobacteria by emulsification using whey proteins as a wall material and following spray drying increased viable counts of bacteria during the storage of yogurt at 4 °C. However, this technique of encapsulation was destructive for microorganisms due to the high temperature of the process [81]. Bifidobacteria microencapsulated in κ-carrageenan were also protected from the low pH of yogurt, and there was no decline of cell number in samples stored for 30 days in the refrigerator. However, consumers preferred yogurt without encapsulated bacteria due to the grainy texture of the fortified one [82,83]. Pinto et al. used gum arabic, inulin, and maltodextrin as wall materials for the encapsulation of *Bifidobacterium lactis* by spray drying. The addition of encapsulated *B. lactis* to yogurt improved its firmness and adhesiveness. However, the encapsulation did not provide additional protection for *B. lactis* [84]. A similar effect was observed for bifidobacteria microcapsules prepared by spray drying using sweet whey or inulin plus sweet whey for encapsulation [85].

The encapsulation of *Lactobacillus paracasei* by the gelation technique using sodium caseinate-gellan gum was found to be a suitable manner for the delivery of bacteria because encapsulated samples showed a higher survival rate than nonencapsulated bacteria. Moreover, the addition of microcapsules and milk protein concentrate improved syneresis and viscosity during the storage of yogurt samples [86]. The microcapsules of the *Lactobacillus acidophilus* strain were prepared by the complex coacervation method using pectin and casein as wall materials [87] and combining ionic gelation and coacervation methods when pectin and whey protein concentrate served as the wall and coating material, respectively [88]. In both cases, the fortification of yogurt with the encapsulated probiotics resulted in the lower values of post-acidification and the higher viability of *Lactobacillus acidophilus* during the cold storage. The microcapsules obtained by the combined method affected negatively the texture of yogurt. It was less acceptable compared to the one with free microorganisms [88]. Despite that, probiotics embedded into alginate beads or chitosan-coated alginate beads were one of the most popular and well-known delivery systems for yogurt [89,90]. Those simple microgels do not ensure the viability of microorganisms exposed to gastric fluids [91]. To increase the protection of bacteria under gastrointestinal conditions, a new approach was developed to produce synbiotic microcapsules. Together with bacteria, prebiotics such as galactooligosaccharides or lactitol were encapsulated in chitosan-coated alginate beads [92]. Although the survival of bacteria enhanced, the quality of yogurt in terms of texture parameters reduced. For yogurt fortification with *Lactobacillus rhamnosus,* protein-based microcapsules were developed using soy protein isolate crosslinked by transglutaminase [93]. The system was found as a suitable alternative to polysaccharide gelation technology. Aljouni et al. showed that whey protein-encapsulated probiotics exhibited increased bioaccessibility in the colon [94].

## 3. Kefir Fortification with Nano/Microencapsulated Bioactive Ingredients

Kefir is one of the oldest fermented milk beverages. It is believed to have originated from the Caucasus region before 2000 BC. Nowadays, kefir is a popular fermented product in Eastern Europe. It is produced by inoculating milk with kefir grains containing a complex mixture of bacteria and yeasts. The flavor and aroma of kefir is the result of metabolic activity and the relationship of a number of bacteria and yeasts species [95].

Kefir is a probiotic drink having human health beneficial effects [96]. However, there are a few publications on the additional functionalization of kefir by the incorporation of encapsulated bioactive components. Yüksel-Bilsel and Sahin-Yesilcubuk developed the functional kefir fortified with encapsulated structured lipids [97]. Microcapsules were prepared by the method of coacervation using gelatin and gum arabic as wall materials. The enzyme, transglutaminase, was used as a crosslinking agent. Fortified kefir was stored within a period of 10 days at 4 °C and remained acceptable in terms of pH value, acidity, and color. Moreover, kefir exhibited no inhibitory effect on oil release in an in vitro digestion study.

Despite the probiotic nature of kefir itself [98], it can be additionally supplemented with probiotics to ensure the sufficient daily consumption of microorganisms. Usually, the encapsulation increases the viability of probiotics during the refrigerated storage and under acid conditions in the digestive tract. That was exemplified by the encapsulation of *Bifidobacterium animalis* ssp. *lactis* BB12 in alginate beads [99] and the preparation of electrospun alginate fibers carrying *Lactobacillus paracasei* KS-199 [100].

The consumption of kefir is growing all over the world. Kefir grains are found all over Europe and beyond. However, nowadays, the popularity of kefir is on the level as the status of yogurts in the 1970s [98]. This fact could explain the limited number of scientific papers on the fortification of kefir by nano/microencapsulated bioactive ingredients. On the other hand, there are a lot of studies concerning health benefits of kefir and its other uses such as the exploitation of kefiran, extracellular polysaccharide produced by LAB [98].

## 4. Cheese Fortification with Nano/Microencapsulated Bioactive Ingredients

### 4.1. Probiotic Microorganisms

For cheese fortification with probiotics, the main challenge is related to the maintenance of probiotic viability during cheese processing and storage. Moreover, the traditional sensory properties of cheese have to be not changed. The survival of bacteria under gastric conditions followed by the colonization of the colon is also very important. Although various factors such as low pH, the presence of salt, oxygen, and the long storage time affect the viability of probiotics, cheese is considered to be a better vehicle for probiotics than yoghurt. It has the higher content of fat and proteins; its dense matrix and good buffering capacity provide greater protection to probiotics [101]. Nevertheless, the survival improvement remains an actual question. Table 3 summarizes the recent research efforts in this direction [102,103,104,105,106,107,108,109,110,111,112,113]. As seen, various types of cheese, soft and hard, are fortified with encapsulated bacteria from the *Lactobacillus* and *Bifidobacterium* genera. Two methods, i.e., extrusion and emulsification, remain the most popular using alginate as a wall material [104,107,108,112]. In addition, the gelation method based on the enzyme action [105,111] or Maillard reaction [103] is used for the fabrication of probiotic carrier.

Usually, the authors observe the survival improvement for the encapsulated probiotics compared to free cells. However, it is quite difficult to estimate the effectiveness of various encapsulation systems and compare them with each other. The investigations are performed using different bacteria strains and different cheese types. The period of time for cheese storage and for the survival evaluation of microorganisms is also different.

### 4.2. Phenolic Compounds (Phenolics) Containing Components

Cheese is a good candidate for the development of functional food. There is a lot of different cheese taste, its shelf life is long, and cheese is a very popular milk product. Having in mind the production on an industrial scale, cheese is fortified mainly by the extracts of plants, but not by pure compounds in order not to increase the production cost. The fortification of cheese with free phenolics can influence the cheese texture, color, and taste during its storage. Moreover, phenolics interact with milk components and lose their antioxidant properties [114]. The extracts of rosemary, fennel, and chamomile were encapsulated by the atomization/coagulation technique using sodium alginate as a coating material. [115,116]. The incorporation of the prepared microcapsules into cottage cheese did not change its nutritional and color parameters. Moreover, the antioxidant activity was maintained more efficiently under cheese storage compared to the free form of extracts. The inexpensive liposomal technology using soy lecithin was developed for the green tea catechins delivery into low-fat and full-fat hard cheese [117,118]. The encapsulation improved the recovery of catechins from digesta in an in vitro gastrointestinal digestion system. However, the recovery of liposomal catechins was significantly dependent on their chemical structure [118,119]. The application of liposomal technology might significantly increase the antioxidant potential of cheese and, overall, the human diet. For the olive phenolics encapsulation and their delivery into white soft cheese, the microcapsules were prepared by ultrasonication and following spray drying. Skim milk proteins and maltodextrin were used as wall materials. The encapsulation ensured invariable antioxidant activity of cheese during 30 days of storage [120]. Pereira et al. showed that quercetin–cyclodextrin inclusion complexes are a suitable approach for fresh cheese fortification with one of the most active flavonoids, which is very sensible to oxidants and light. [121]. γ-Cyclodextrin was found to be the better host molecule than β-cyclodextrin due to the larger interior cavity and deeper guest inclusion. At the concentration of 0.03–0.04% (*w*/*w*), quercetin–cyclodextrin inclusion complexes resulted in the minor organoleptic changes such as the firmer texture and yellowish color.

Taken together, the scientific publications on the fortification of cheese with nano/microencapsulated phenolics are not numerous.

### 4.3. Other Bioactive Components

For cheese, carotenoids serve as health beneficial compounds or colorants [14,122]. Recently, the investigation on the supplementation of Appezeller cheese and Queso Blanco cheese with microencapsulated tomato extract as a source of lycopene has been performed. For the fabrication of microcapsules, the emulsion was spray-dried using maltodextrin as a coating material. Sensory properties of cheese were improved due to tomato taste and higher yellowness [123,124]. The pepper extract rich in carotenoids and polyphenols was encapsulated in alginate beads by the ionic gelation method and used for the production of a new Labneh cheese [125]. For the production of Cheddar and Gouda cheeses, the colorant annatto responsible for the yellowish-red color is used. Annatto is extracted from the seeds of the *Bixa orellana* plant and is composed of the two major carotenoids, norbixin and bixin [122]. However, during the manufacturing process, the small amount of annatto (5–10%) is transferred to a whey stream and reduces the quality of whey powder. Moreover, there are regulatory restrictions concerning the amount of annatto in whey, especially for the application in infant formula [126]. To reduce the amount of colorant in whey powder, a new alternative based on the encapsulation of annatto was proposed. The colorant was complexed with chitosan [127,128] or entrapped into casein–chitosan complexes [129]. Moreover, enzyme-responsive microcapsules that can selectively color the cheese matrix were developed [130].

There are a few works aimed at the delivery of omega-3 originated from animal and vegetables sources to cheese. Chia seed oil is rich in α-linolenic acid. Sheep’s milk cheese was fortified with chia oil emulsion stabilized by calcium caseinate. The enrichment did not affect the cheese-making process and microbial quality during the ripening period [131]. Double emulsions of canola oil rich in omega-3 were prepared by ultrasonication and displaced in Cheddar cheese [132]. Cod liver oil emulsions stabilized by self-assembly structures based on saturated monoglycerides ensured the good retention of omega-3 source in the cheese curd. The texture parameters were similar to unfortified fresh soft cheese [133]. Stratulat et al. prepared the flaxseed oil emulsion stabilized by calcium caseinate. That emulsion was not only rich in α-linolenic acid but also served as a carrier of vitamin D_3_ for the delivery into cheese [134]. In addition, vitamin D_3_ can be delivered in the form of liposomes [135]. Flaxseed oil-based emulsion can also be used for the encapsulation of coenzyme Q_10_ or coencapsulation with vitamin E and A [136,137]. As a result, the retention of the lipophilic compounds in the curd increased, and their stability and functionality were also improved during cheese storage.

For hydrophilic vitamin B_12,_ double emulsion (water-in-oil-in-water) was developed using butter oil, lipophilic emulsifier polyglycerol polyricinoleate, and hydrophilic emulsifier sodium caseinate [138]. The encapsulation improved the retention of vitamin B_12_ in the cheese curd as compared to the nonencapsulated vitamin and increased its stability during in vitro gastric digestion. Cheese could be a suitable vehicle for iron supplementation due to its popularity and large consumption. However, the fortification of cheese with microencapsulated ferrous sulfate negatively affects its organoleptic properties, and the microencapsulation is unable to mask the iron taste, color, and odor [139].

Bioactive compounds are incorporated into cheese to increase its health benefits. However, some of them can serve as a natural preservative. This is very important for soft, fresh cheese having the high level of moisture. Lemongrass essential oil composed mainly of α-citral and β-citral was encapsulated into microparticles by the preparation of emulsion and the following spray drying using Arabic gum and maltodextrin as wall materials. The obtained microcapsules were effective in controlling the proliferation of microorganisms in Coalho cheese during 21 days of storage [140]. The encapsulated rosemary essential oil at the concentration of 0.5% delayed microorganism growth in Minas frescal cheese. Microcapsules were prepared by spray drying using inulin and whey protein isolate as wall materials [141].

## 5. Conclusions

Intensive research is underway for increasing the functionality of popular fermented milk products. Despite the health benefits of fermented milk products themselves, research is stimulated by the growing consumer interest in healthy foods. Kefir, yogurt, and cheese are appropriate vehicles for various bioactive ingredients such as carotenoids, phenolic compounds, probiotics, omega-3, and other micronutrients. The encapsulation serves for the enhancement of stability and solubility of bioactive compounds or the survival improvement of probiotic microorganisms during product processing, storage, or undergoing gastric digestion. The analysis of scientific papers shows that with some exceptions, the delivery system is proposed for each core material separately. Further research could be aimed for the development of more complex delivery systems allowing the fortification of fermented milk products at once with several different additives. In some cases, the coencapsulation can be complicated due to the different and not always compatible physicochemical properties of bioactive ingredients.

## Figures and Tables

**Table 1:** Encapsulation of carotenoids and carotenoids containing components for yogurt’s fortification.

Bioactive Component	Encapsulation Techniques and Systems	Encapsulant Materials	Observations	References
Tomato peel extract	Electrospinning	Zein, gelatin	Similar properties to control yogurt sample in terms of yogurt acidity, pH, syneresis, and viscosity. The increase of yogurt free radical scavenging activity by 40–60%.	[18]
Red pepper waste extract	Freeze drying, spray drying	Whey proteins	Fortified yogurt showed higher sensory and general acceptability scores compared to control sample.	[19]
Red bell pepper extract	Inclusion complexation by ultrasonic homogenization	β-Cyclodextrin	A mean particle diameter was 562 nm. The prepared complexes can simulate the color of papaya flavors. The higher stability of color was observed for yogurt colored with inclusion complexes comparing with yogurt colored with crude extract.	[20]
Cantaloupe melon extract	Emulsification in O/W followed by lyophilization	Gelatin as a wall material	The solubility of extract encapsulated in gelatin (EG) was 0.072 mg/mL. EG gave homogenous yellow coloration to yogurt; staining was preserved over 60 days.	[21]
β-Carotene	Spray-dried emulsion followed by fluidized bed coating	Maltodextrin or sodium caseinate for emulsification and hydroxypropyl cellulose for coating.	Coated powders were used for yogurt coloration. After 4 weeks of storage, the changes of yogurt color were minimal. Total color difference ΔE value was < 3.	[22]
β-Carotene	Multilamellar liposomes obtained by the hydration of proliposomes	Stabilized by the mixture of xanthan and guar gums.	About 90% of the encapsulated β-carotene was preserved after the storage for 95 days at 7–10 °C. Liposomes were tested as colorant in yogurt. Its texture was not affected by the incorporation of the liposomes.	[23]
β-Carotene	Solid lipid microparticles (SLM)	Palm stearin as the lipid phase and hydrolyzed soy protein isolate for particles stabilization	The average diameter of SLM was 1.2 µm. The amount of 25 g (500 mL) of SLM was added to 10 L of yogurt. The addition of SLM did not change the physicochemical and rheological characteristics of yogurt. Based on sensory analysis, the average grade of global acceptance was 7.4 (“liked it very much”) on the hedonic scale.	[24]
β-Carotene	Spray drying; coacervation for beads	Maltodextrin for spray drying Chitosan and alginate for beads	In yogurt, spray-dried β-carotene showed higher release and the incorporation into micelle phase during digestion than β-carotene encapsulated in chitosan–alginate beads.	[25]
Zeaxanthin	Nanoemulsion by high-pressure homogenization	Zeaxanthin was dispersed in chia seed oil. Tween 80 for emulsification.	After 28 days of yogurt storage, zeaxanthin retention was 16.84%. Higher bioaccessibility as compared to zeaxanthin nanoparticles.	[26]
Zeaxanthin	Nanoparticles	Cactus mucilage as a wall material. Zeaxanthin was dispersed in chia seed oil.	After 28 days of yogurt storage, zeaxanthin retention was 22.31%. The incorporation of zeaxanthin nanoparticles decreased the texture and viscosity and increased the syneresis when compared to the control yogurt. The changes were not sensory perceived.	[26]
Astaxanthin	Beads prepared using ultrasonic atomizer	Alginate and chitosan	The concentration of beads in yogurt was 15% (*w*/*w*). No differences were observed for the appearance, color, and aroma as compared to control yogurt. Consumers perceived differences in terms of taste, mouthfeel, and overall liking attributes.	[27]
Lipid extract of astaxanthin from shrimp waste	Complex coacervation with following freeze drying.	Gelatin and cashew gum	Coloring capacity of microcapsules was compared to non-encapsulated lipid extract. Both forms of astaxanthin yielded the orange color of yogurt. Encapsulated form showed more intense color. No differences in odor between the yogurt sample containing encapsulated lipid extract and the sample containing non-encapsulated lipid extract were found.	[28]
Palm oil	Coacervation	Chitosan and carboxymethylcellulose	Particles showed the ideal carotenoid release in gastric fluid, but low release in the intestinal fluid, which increased when applied to yogurt.	[29]
Palm oil	Ionic gelation	Chitosan plus sodium tripolyphosphate as a cross-linker	Particles showed high carotenoid release in gastric fluid, but satisfactory release in intestinal fluid. The release further increased when the particles were added to yogurt.	[29]
Palm oil	Coacervation followed by lyophilization	Chitosan and xanthan; chitosan and pectin	Chitosan/xanthan microparticles applied in yogurt released approximately 50% of the content in the intestinal fluid. The behavior of release was similar to the desired one. The release of content from chitosan/pectin microparticles was slower.	[30]

**Table 2:** Encapsulation of phenolic compounds containing components for yogurt’s fortification.

Bioactive Component	Encapsulation Techniques and Systems	Encapsulant Materials	Observations	References
Grape seed extract	Spray drying	Whey proteins and gum arabic	The addition of encapsulated grape seed extract at the final concentration of 1% resulted in similar sensory properties, viscosity, acidity, water-holding capacity, and color compared to the control. The antioxidant activity increased four-fold.	[34]
Orange peel extract	Coacervation	Whey proteins and gum arabic	The negative influence on the physicochemical and organoleptic properties of yogurt was not observed.	[39]
Sour cherry extract	Spray-dried coated liposomes	Lecithin for liposomes preparation and chitosan for their coating.	Liposomal powders (LP) were added to yogurt with a ratio of 5% (*w*/*w*). The addition of LP did not change the color parameters of yogurt up to 14 days of storage. Encapsulation provided the stability of extract in terms of total phenolic content and antioxidant capacity.	[35]
Doum extract	Coated liposomes	Lecithin for liposomes preparation and chitosan for their coating.	Naringenin was the main component of extracted phenolics. Yogurt fortified with 5% of liposome solution had similar characteristics including the acidity, water-holding capacity, and texture parameters as a control yogurt, but the antioxidant activity was higher. The addition of higher percentage of encapsulated product affected markedly the functional properties of yogurt.	[36]
Cocoa hull waste extract	Spray-dried coated liposomes	Lecithin for liposomes preparation and chitosan for their coating.	For yogurt fortification, liposomes in two forms, i.e., dispersion and sprayed-dried powders, were used. The best results were found for the powder form in terms of total phenolic compounds and total antioxidant activity.	[37]
Hibiscus calyx extract	Double emulsion and following ionic gelation.	Rapeseed oil and pectin	The extract is rich in anthocyanins. Microparticles were obtained by dripping-extrusion and atomization methods. The microparticles were added to yogurt with a ratio of 20% (*w*/*w*). The yogurt supplemented with microparticles obtained by atomization had higher appearance acceptability, but the retention of bioactive compounds was lower comparing to dripping techniques.	[40]
Tartary buckwheat extract	Beads were prepared by thermally-induced polymerization of proteins and following spray drying.	Whey proteins	The extract is rich in rutin and quercetin. Yogurt contained 3% (*w*/*w*) of beads. Encapsulation masked the dark yellow color and bitter taste of extract and protected flavonoids from the gastric juice.	[45]
Olive leaves extract	Nanoliposomes	Lecithin and cholesterol with the ratio of 4:1.	The extract is rich in oleuropein. The amount of 100 g yogurt was fortified with 15 g (containing 10% of phenolics) of nanoliposomes. No changes in color and sensorial attributes were observed. The antioxidant activity did not change during the yogurt storage for 21 days.	[44]
Eggplant (*Solanum melongena* L.) bark extract	Spray drying	Gum arabic	The extract is rich in anthocyanins. The amount of 1, 1.5, and 2 g of encapsulated and nonencapsulated extract was added to 100 g of yogurt. More than 50% of free anthocyanins degraded in yogurt after 20 days of storage, whereas encapsulated ones remained stable. The decrease of antioxidant activity was slower for yogurts fortified with the encapsulated form of extract.	[38]
Date palm pollen	Nanocapsules obtained by ultrasonication	Sodium caseinate and lecithin	The extract is rich in catechin. The size of capsules was approximately in the range of 200–300 nm. The amount of pollen extract in free and encapsulated form was 0.75% (*w*/*v*) of milk. No color changes were observed for yogurt fortified with encapsulated pollen extract. It also scored the higher scores of appearance and body and texture compared to yogurt fortified with free extract. Overall, sensorial acceptability was also higher.	[41]
Resveratrol	Niosomes	Surfactants sorbitan monostearate (S60), labrasol (Lab) and maisine 35-1(Mai), and lauryl alcohol (Dod) as stabilizer	Niosomes were prepared by thin film hydration method. About 10% (*v*/*v*) of niosomes suspension was added to yogurt. The texture parameters (firmness and adhesiveness) of yogurt fortified with Mai-Dod and S60-Dod niosomes were the same as of control ones. Yogurt enriched with Lab-Dod niosomes showed the decrease in the adhesiveness.	[42]

**Table 3:** Encapsulation of probiotics for cheese fortification.

Cheese Type	Microorganism	Encapsulation Techniques and Systems	Encapsulant Materials	Observations	References
Cream cheese	*Lactobacillus rhamnosus*	Microgel particles obtained by aerosol spraying	Sodium alginate	*L. rhamnosus* remained viable (min 10^6^ CFU/g) over 35 days of storage at 4 °C. The addition of encapsulated probiotic bacteria resulted in a firmer and thicker cheese.	[102]
White brined cheese	*Lactobacillus rhamnosus*	Microcapsules obtained by cold gelation	Maillard reaction products of isomaltooligosaccharides and whey proteins	Droplet-like capsules with a smooth surface, whose diameter was approximately 183 μm. The viability of encapsulated probiotic was increased compared to free cells during 90 days of storage at 4 °C. Sensory properties of cheese were not affected by the addition of encapsulated bacteria.	[103]
White brined cheese	*Bifidobacterium bifidum*, *Lactobacillus acidophilus*	Extrusion, emulsion	Sodium alginate for extrusion and corn oil and κ-carragenan for emulsion	Both techniques were effective in keeping cell counts higher than the therapeutic minimum during 90 days of storage at 4 °C. The content of free fatty acids, acetaldehyde, and diacetyl was higher as compared to control sample.	[104]
Iranian UF Feta cheese	*Lactobacillus paracasei*	Enzyme based gelation (rennet or transglutaminase)	Sodium caseinate or skim milk powder	The cells entrapped into microcapsules by rennet-based gelation exhibited the higher viability compared to transglutaminase-based gelation.	[105]
Iranian UF white cheese	*Lactobacillus plantarum*	Complex coacervation followed by spray drying or freeze drying	Whey protein isolate and gum arabic	Phytosterol was coencapsulated with bacteria. Its coencapsulation increased the viability of bacteria in cheese during 91 days of storage at 4 °C in comparison with encapsulated bacteria alone or free cells.	[106]
Iranian white brined cheese	*Lactobacillus acidophilus*	Extrusion	Calcium alginate and resistant starch	The survival of bacteria was increased in cheese and was equal to ≥10^7^ CFU/g after 6 months of storage.	[107]
Iranian UF white cheese	*Lactobacillus plantarum,Bifidobacterium bifidum, Lactobacillus casei* subsp. *casei*	Extrusion	Sodium alginate	After 60 days of cheese storage at 8–10 °C, the counts of each encapsulated bacterium were higher than the therapeutic minimum (10^6^–10^7^ CFU/g).	[108]
Cheddar cheese	*Bifidobacterium bifidum*	Emulsification/internal gelation	κ-carrageenan or sodium alginate	For encapsulated bacteria, the decrease of viability was slower over a period of 35 days. The encapsulant sodium alginate showed the better results than κ-carrageenan under simulated gastrointestinal conditions.	[109]
Cheddar cheese	*Bifidobacterium longum*	Co-axial droplet extrusion, emulsification/internal gelation	Sodium alginate or palmitoylated alginate	After 21 days of cheese storage at 4 °C, bacteria encapsulated by emulsification/internal gelation showed 2 log CFU/mL reduction as compared to free cells with 4 log CFU/mL reduction.	[110]
Kariesh cheese	*Bifidobacterium adolescentis*	Rennet based gelation	Milk proteins	The viability of encapsulated bacteria increased during two weeks of cheese cold storage.	[111]
Mozzarella cheese	*Lactobacillus paracasei*	Emulsification/internal gelation	Sodium alginate	Encapsulation provided no protection against simulated gastric juice.	[112]
Soft goat cheese	*Lactobacillus plantarum*	Spray drying	Skim milk	After 8 weeks of cheese storage, the high level of 8.82 log CFU/g was found for the encapsulated bacteria, while the free-cell number decreased to 6.9 log CFU/g. The addition of spray-dried bacteria did not change the properties of cheese (pH value, chemical composition, sensory quality).	[113]

## Data Availability

The data presented in this study are available on request from the corresponding author.

## Sample Availability

Samples of the compounds are not available from the authors.
